# Current Knowledge on Functionality and Potential Therapeutic Uses of Donkey Milk

**DOI:** 10.3390/ani11051382

**Published:** 2021-05-13

**Authors:** Mina Martini, Iolanda Altomonte, Domenico Tricò, Riccardo Lapenta, Federica Salari

**Affiliations:** 1 Department of Veterinary Science, University of Pisa, 56124 Pisa, Italy; mina.martini@unipi.it (M.M.); r.lapenta@studenti.unipi.it (R.L.); federica.salari@unipi.it (F.S.); 2 Interdepartmental Center for Agricultural and Environmental Research “E. Avanzi,”, University of Pisa, San Piero a Gardo (PI), 56122 Pisa, Italy; 3 Department of Surgical, Medical and Molecular Pathology and Critical Care Medicine, University of Pisa, 56100 Pisa, Italy; domenico.trico@unipi.it

**Keywords:** donkey milk, human health, milk composition, milk whey protein, milk fat, food allergies, immunomodulatory properties, cancer, intestinal microbiota, oxidative stress, dyslipidemia

## Abstract

**Simple Summary:**

This paper examines scientific evidence on the positive effects of donkey milk consumption on human health and its possible therapeutic applications. The most investigated clinical use of donkey milk is in feeding infants with food allergies, in whom donkey milk is well tolerated in the 82.6–98.5% of cases. Donkey milk has shown several beneficial properties, including immunomodulatory activity, antioxidant and detoxifying effects, modulation of the intestinal microbiota, and lowering of blood sugar and triglycerides, which have been tested almost exclusively in experimental animals. Inhibitory actions on microorganisms have been also observed in vitro studies. This literature review highlights the need for new clinical trials to collect stronger evidence about the positive effects observed in experimental models which could lead to new therapeutic applications of donkey milk in humans.

**Abstract:**

The increase of knowledge on the composition of donkey milk has revealed marked similarities to human milk, which led to a growing number of investigations focused on testing the potential effects of donkey milk in vitro and in vivo. This paper examines the scientific evidence regarding the beneficial effects of donkey milk on human health. Most clinical studies report a tolerability of donkey milk in 82.6–98.5% of infants with cow milk protein allergies. The average protein content of donkey milk is about 18 g/L. Caseins, which are main allergenic components of milk, are less represented compared to cow milk (56% of the total protein in donkey vs. 80% in cow milk). Donkey milk is well accepted by children due to its high concentration of lactose (about 60 g/L). Immunomodulatory properties have been reported in one study in humans and in several animal models. Donkey milk also seems to modulate the intestinal microbiota, enhance antioxidant defense mechanisms and detoxifying enzymes activities, reduce hyperglycemia and normalize dyslipidemia. Donkey milk has lower calorie and fat content compared with other milks used in human nutrition (fat ranges from 0.20% to 1.7%) and a more favourable fatty acid profile, being low in saturated fatty acids (3.02 g/L) and high in alpha-linolenic acid (about 7.25 g/100 g of fat). Until now, the beneficial properties of donkey milk have been mostly related to whey proteins, among which β-lactoglobulin is the most represented (6.06 g/L), followed by α-lactalbumin (about 2 g/L) and lysozyme (1.07 g/L). So far, the health functionality of donkey milk has been tested almost exclusively on animal models. Furthermore, in vitro studies have described inhibitory action against bacteria, viruses, and fungi. From the literature review emerges the need for new randomized clinical trials on humans to provide stronger evidence of the potential beneficial health effects of donkey milk, which could lead to new applications as an adjuvant in the treatment of cardiometabolic diseases, malnutrition, and aging.

## 1. Introduction

Donkey milk (DM) has been historically considered a therapeutic food in both Western and Eastern cultures. Hippocrates (460–370 BC) [1] and Pliny the Elder (23–79 AD) [2] were among the first to describe DM health benefits. The beneficial properties of DM are also reported in traditional Chinese medicine manuals [3].

The studies of Dr. Parrot of l’Hospice des Enfants Assistès (Paris, France) in the nineteenth century were probably the first scientific approach to the use of DM in infant feeding. Dr. Parrot fed children affected by congenital syphilis directly from the donkey’s udder. In particular, Dr Parrot carried out the first controlled trials on breastfeeding with DM, comparing DM with cow and goat milk, recording the milk intakes, the weight gains of the children, and analysing DM chemical composition. Dr Parrot’s studies led to the creation of a donkey farm for the purpose of feeding orphaned children [4].

In recent decades, alongside with the rediscovery of its potential beneficial effect, DM is becoming popular in Europe, especially in Croatia, France, Hungary, Italy, the Netherlands and Serbia, and in several Asian countries. In particular, China is a large donkey meat and milk producer, and the donkey industry has become important in rural China. 

DM is considered the natural milk with the closest composition to human milk in terms of lactose content and protein and amino acid profile [5]. 

Scientific studies aimed at clarifying the composition of DM and the presence of functional compounds have increased over the past years. Until now, investigations have been carried out to highlight potentially bioactive substances, such as polyunsaturated and omega 3 fatty acids [6,7], functional proteins [8,9], vitamins [10,11], polar lipids [12], phytosterols [13], and the milk compositional variability [14,15].

New knowledge has emerged leading to the development of studies focused on testing in vitro and in vivo the potential effects of DM in humans. This paper examines the scientific evidence regarding the effects of DM on human health and its possible applications as an adjuvant in the treatment of cardiometabolic diseases, malnutrition, and aging. 

## 2. Use of DM in Allergic Children

DM has nutritional similarities with human milk (HM), the gold standard for infant feeding, in terms of average protein content (about 18 and 21 g/L in DM and HM, respectively) [5]. Caseins (CN), which are main allergenic components of cow milk (CM), are less represented in DM and HM (56% and 30% of the total protein in DM and HM vs. 80% of CM). A major similarity is also linked to the primary structures of αS1-, β- and κ-CN, which are closely related in the HM and DM [16]. The main CN fraction of DM is β-CN (about 6.11 g/L; 62% of total CN), whose concentration is between the minimum values found in HM (1.25–4.72 g/L) and the maximum in CM (11.85–12.87 g/L). The mean concentration of αS1-CN in DM is 2.54 g/L (26% of total CN), higher than HM (0.33–0.50 g/L) and lower than in CM (8.52–9.16 g/L); αS2-CN and κ-CN are minor component of DM [9].

Simulated in vitro digestion showed that donkey CN has rapid degradability and an almost complete digestibility both when artificial [17,18] and gastrointestinal fluids human fluids [19,20] were used.

This can also explain the reduced allergenicity of DM since food protein allergenicity is linked to the survival of allergens in the gastrointestinal tract.

On the other hand, the main DM protein fraction is made up by whey proteins (WP), which have shown multiple beneficial metabolic and antimicrobial properties [21]. β-Lactoglobulin (β-LG) is the most represented WP (6.06 g/L; 73% of total WP), absent in HM but present in CM in a concentration of about 5 g/L. The α-lactalbumin (α-LA) content in DM (about 2 g/L) is similar to HM (3 g/L) [9].

The similarities between HM and DM are at the basis of a well-known application of DM: its use in the diet of children suffering from allergies to CM proteins (CMPA). In this regard, most clinical trials on humans proved the tolerability and efficacy of DM in these patients (Table 1).

Most trials in the literature are single-arm prospective longitudinal studies [22,23,24,25], while there are still few randomized studies involving a control group [26]. The papers available so far have included a limited number of subjects (<100 enrolled patients) with a rather wide age range, on average between 27 months and five years [24,25], suffering from CMPA, food protein-induced enterocolitis syndrome induced by cow milk (CM-FPIES), and multiple food allergies.

The reported tolerability of DM in allergic children varies between 82.6% [23] and 98.5% of patients [22]. In addition, a clinical study in infants under six months of age, conducted on a low number of subjects (six infants) with CM-FPIES, has found that DM is well tolerated also in younger patients, with no participants showing allergic reactions [27].

Even though encouraging results support the consumption of DM in children with food allergies, some cases of hypersensitivity reactions to DM have been reported in children and in two adults suggesting caution in allergic subjects [28,29,30,31].

**Table 1:** Studies on the tolerability of donkey milk in children.

Study Design	Number of Children	Mean Age	Duration of Diet	Tolerance Outcome	Reference
Double-blind placebo-controlled food challenge	30 with the IgE- and non-IgE-mediated CMPA ^1^	2.5 years (from 0.6 to 3.8 years)	3 months	96%	[26]
Prospective study; double-blind, placebo-controlled food challenge	46 with IgE- and non-IgE-mediated CMPA	36 months (from 12 to 149 months)	24 months	82.6% of the total patients (78.8% of the children with IgE-mediated CMPA)	[23]
Prospective study	92 highly-problematic children with IgE- and the non-IgE-mediated CMPA	27.3 months (from 7.5-to 121.5 months)	48 months	87% children with non-IgE-mediated CMPA (20/23) 91.3% with IgE-mediated CMPA. (63/69)	[24]
Open challenge	70 children including patients with prior anaphylaxis to CM ^3^	5.2 ± 5.3 months (from 6 months to 18 years)	/	98.5%	[22]
Open challenge	70 children with proven IgE-CMPA; 11 patients with proven IgE-FPIES	5.2 ± 5.3 years (from 6 months to 18 years); 4.73 ± 1.68 months (from 3 to 8 months)	/	98.7%	[25]
Open challenge	6 with CM-FPIES ^2^	3.6 months (from 1.5 to 6 months)	/	100%	[27]
Open challenge	30 with IgE- and non-IgE-mediated CMPA	4,5 years (from 6 months to 11 years)	/	96%	[31]

^1^ CMPA: cow milk protein allergy; ^2^ CM-FPIES: food protein-induced enterocolitis syndrome induced by cow milk; ^3^ CM: cow milk.

From the point of view of palatability [23,25], DM is well accepted by the children. The good palatability is probably related to the high concentration of lactose [5] and to the fact that allergic children often follow restrictive nutritional plans and monotonous diets due to multiple food allergies. 

Regarding nutritional efficacy, research shows that (Table 2), despite the majority of allergic subjects have negative weight and length/stature Z-scores due to feeding difficulties, growth parameters improve after DM supplementation [23,24,26,31]. The positive effect of DM on growth is probably related to the ability of the milk to fill some nutritional gaps in the diet of treated subjects [26]. Even in infants under one year of age, if properly integrated, DM did not show negative effects on the growth [25]. 

Nonetheless, the fat percentage of DM is usually three times lower than HM and CM, and consequently its energy content is also lower (about 40 vs. 62 and 65 kcal/100 g, respectively) [5]. According to nutritional recommendations of different international organizations, fat should provide 40–60% of the daily energy intake in pre-weaned children (between 0 and 6 months) and should be gradually reduced to 35% in children 2 years old [32]. Therefore, since DM has a lower fat and caloric content compared to other milks used in infant feeding, fat needs to be supplemented in children consuming an exclusively milky diet, e.g., by adding vegetable oils.

The Diagnosis and Rationale for Action against Cow’s Milk Allergy (DRACMA) guidelines report that equine milks can be considered valid substitutes for CM even if they cannot be a treatment of choice for CMPA [33]. The choice of alternative milks should take into account the individual clinical profile of the child allergic to CM, particularly as concerns age, severity of symptoms, sensitivity to CM proteins and associated food allergies [34]. 

Recently, a line of research regarding the application of DM as a fortifier in the feeding of premature newborns has developed [35,36]. Human milk fortification is a routine clinical practice for feeding preterm infants to ensure protein and energy intakes of critical importance in preterm infants. 

To evaluate the effect of HM fortification with DM compared to CM, Bertino et al. [35] designed an open label, randomized controlled clinical trial on a total of 156 very preterm newborns (gestational age <32 weeks; very-low-birthweight <1500 g) giving two isocaloric and isoproteic diets. The authors found that a DM-based fortifier seems to improve feeding tolerance, with a similar auxological outcome in the first 21 days of enteral diet compared to a CM-based fortifier. A follow-up analysis on 122 of these children at 18 months of age reported that the fortifier derived from DM have similar long term auxological outcomes compared with the standard CM-derived fortifier [30]. 

Finally, ancillary studies of Bertino et al. [35] and Peila et al. [37] have also shown that the DM compared to CM fortifier reduced the episodes of gastroesophageal reflux (GER), which frequently occur in very-low-birthweight infants [36]. GER is worsen by food intolerance and can be associated with cardiorespiratory symptoms [38]. In the paper by Cresi et al. [37], very preterm infants taking DM also had a lower frequency of weakly acidic reflux (characteristic of GER) compared to the control group. Given the lower buffering capacity of DM compared with CM, DM did not affect the physiological acid reflux. The authors [37] state that DM minor buffering effects on gastric pH could be a protective factor, preventing infections and necrotizing enterocolitis in premature infants.

**Table 2:** Studies on the effects of donkey milk on the growth of infants and children.

Study Design	Number of Children	Age	Diet	Auxological Outcome	Reference
Prospective study	16 with IgE-CMPA ^1^ and 6 CM-FPIES ^2^	20 months (range 9–79 months).	Integrated with DM for 6 months	No negative influence	[25]
Randomized controlled trial	156 preterm infants (77 assumed DM ^3^–fortifier)	11 days (median age)	DM- fortifier vs. CM ^4^- fortifier; isocaloric and isoproteic diets for 21 days	Similar auxological outcomes than control group	[35]
Randomized controlled trial	122 children (77 assumed DM fortifier)	18 months	DM–derived fortifier vs. CM fortifier	Similar auxological outcomes than control group	[37]

^1^ CMPA: cow milk protein allergy; ^2^ CM-FPIES: food protein-induced enterocolitis syndrome induced by cow milk; ^3^ DM: donkey milk; ^4^ CM: cow milk.

## 3. Immunomodulatory Effects 

DM is particularly rich in lysozyme (LZ) [8], an enzyme that breaks the peptidoglycan layer of Gram-positive bacteria. The average LZ concentration of DM is on the average 1.07 g/L (13% of total WP) [9], similar to HM (0.3–1.1 g/L; [39]) and higher than CM, in which negligible amounts of LZ have been reported. LZ activity for DM ranges from 1670 to 11,531 U/mL [8,40], while it is barely detectable in CM (0.0292 U/mL) and the highest in HM (about 39,000 U/mL) [41].

According to by Mao et al. [42], the WP fractions containing LZ are responsible for DM immunomodulatory effect. Whether other components contribute to this action, such as α-lactalbumin (α-LA) and lactoferrin, remains to be determined. In fact, α-LA seems to regulate the overall immune function infants [43]. Recently, investigations on milk oligosaccharides have also shown positive effects on immune system development. However, research on oligosaccharides regards mostly HM, whereas DM is less studied [44].

DM have shown immunological activities in vitro tests [42,45] and randomized controlled studies in animal models [46] and humans [47], in whom DM can induce the release of some cytokines, proteins that regulate the inflammatory and immune response to infections (Table 3 and Table 4). DM has been shown to increase cytokines involved in the regulation of innate immunity and the onset of local acute inflammatory response: interleukin 1 (IL-1) [42,45,47], interleukin 6 (IL-6) [42,47] and tumor necrosis factor α (TNF-α) [42,47] both in vitro [42] and in vivo [47]. Differently, Jiang et al. [46] have reported an inhibition of TNF-α in mice with inflammatory bowel disease (IBD).

Furthermore, the WP fraction of DM with a molecular mass >10 kDa has been shown to stimulate the production of specific immunity regulatory cytokines such as interleukin-2 (IL-2) and interferon γ (IFN-γ) by murine splenocytes [42]. DM has also induced the release of interleukin-10 (IL-10), which is responsible for reducing inflammatory reactions, helping the elimination of pathogens and reducing the infection damage [47].

The inhibition of the expression of inflammation mediators, in particular interleukin 13 (IL-13) and the already-mentioned TNF-α, has been observed by Jiang et al. [46] on mice with inflammatory disease.

The only study concerning the immunomodulatory effects of DM in humans has involved elderly subjects (14 healthy aged subjects; from 72 to 97 years), in which DM vs. goat milk was administered [47]. These authors observed that the administration of DM (200 mL/day for one month) acts as an enhancer of the acute phase response in humans. Therefore, DM daily use may be recommended in the diet of immuno-compromised elderly patients. 

## 4. Potential Antioxidant and Antihypertensive Effects

The antioxidant activity of DM has been tested in double-blind randomized studies on animal models [17,48,49] (Table 4). DM-treated rats have shown an enhancement in antioxidant defense mechanisms and detoxifying enzymes [17,48,49].

Specifically, Li et al. [17] found that DM intake tended to increase the superoxide dismutase (SOD) activity in the plasma of diabetic rats compared to untreated rats. SOD enzyme alternately catalyzes the dismutation (or partitioning) of the superoxide (O_2_^−^) radical into ordinary molecular oxygen (O_2_) and hydrogen peroxide (H_2_O_2_). Furthermore, Li et al. [17] showed that total anti-oxidation capacity is also improved in diabetic rats treated with DM compared with the untreated group, towards values seen in healthy (control) rats.

In mouse models, improvements in glutathione/glutathione disulfide ratio in liver (i.e., an oxidative stress marker) were also observed, as well as increased activities of liver detoxifying enzymes (glutathione-S-transferase—NAD (P) H: Quinone Oxidoreductase) [48,49].

Antioxidant activities directly measured in DM and fermented DM (kefir) by mean of ABTS (2, 2′-Azino-bis (3-ethylbenzothiazoline-6-sulphonic acid) and DPPH (2, 2 diphenyl-1- picryl hydrazyl) assays have been found higher in kefir than raw DM and increased after in vitro simulated gastrointestinal digestion [18]. A role of fermentation in DM antioxidant activity has also been ascribed to bacteria, in particular *Enterococcus faecium* DM33, as fermented milk containing this bacterium exhibited the strongest antioxidant activity [18].

However, antioxidant activities could be related to the release of bioactive peptides through the enzymatic hydrolysis of proteins. A peptidomic study [50] identified 1330 peptides from commercial donkey milk, mainly coming from β-CN, αS1-CN and serum amyloid A protein. Moreover, β-LG I and lactoferrin can be source of milk peptides, while α-LA and LZ are resistant to gastrointestinal enzymes [16]. DM peptide fractions tested by in vitro bioassays have shown antioxidant activities [19,51].

Many peptides in DM have typical characteristics of angiotensin-converting enzyme (ACE)-inhibitory peptides, potentially reducing the activity of ACE [16]. DM fractions containing different peptides confirmed angiotensin converting-enzyme inhibitory actions when tested by in vitro bioassays [50,51].

None of the potentially bioactive peptide identified in DM by Zenezini Chiozzi et al. [51] exactly matches sequences of known bioactive peptides. However, potential ACE inhibitory peptides (namely MPFLKSPIVPF) had a similar sequence and the same length to a confirmed antihypertensive peptide (namely MPFPKYPVQPF) which was previously found in Gouda cheese.

Therefore, milk-derived bioactive peptides may potentially decrease the formation of angiotensin II and increase bradykinin levels, which have vasoconstrictor and vasodilator properties, respectively. The actions act synergistically in lowering blood pressure. In this regard, fermented DM (with *Lactobacillus casei* DM214) showed ACE-inhibitory activity in vitro [18]. 

In addition, the release nitric oxide (NO) from peripheral blood mononuclear cells (PBMCs) treated with DM was observed in vitro. Since nitric oxide (NO) is a strong vasodilator, a role for DM in the prevention of atherosclerosis has been proposed [45]. 

## 5. Effects on Glucose Metabolism and Potential Coadjutant Action in the Diabetes Treatment

Lactose is the main carbohydrate in DM, as in HM and CM (about 70, 60 and 49 g/L of milk respectively) [5,41] and it is responsible for the osmotic equilibrium between blood and alveolar lumen in the mammary gland. Lactose can assume two anomeric forms (α-lactose, and β-lactose) on the basis of the glycosylic bond (1,4) that connects the carbon atom 1 of galactose and the carbon atom 4 of glucose [52].

Lactose intolerance is common in the adult population mostly due to the loss of intestinal lactase; its prevalence has great geographical variability [53]. Many intolerant individuals can tolerate low levels of lactose in their daily diet (about 5–10 g of lactose distributed throughout the day) and in general the use of fermented products and of lactase supplements can overcome the problem [52]. To the best of our knowledge, there are no specific studies regarding the use of DM in subjects with lactose intolerance. Lactose-free DM is currently not available on the market.

Lactose, WP, and bioactive peptides in DM have been shown to be involved in insulin response to glucose [54]. In particular, LZ and α-LA from DM may have a role in the prevention and treatment of diabetes [17]. α-LA is a highly represented protein in DM (on the average 1.22 g/L; 14.69% of total WP) and HM (2.6–4.2 g/L) and plays a key role in lactose synthesis in the mammary gland.

Beneficial glucometabolic properties of DM have been observed in animal studies [17,48,49]. However, the active components of DM and the biological mechanisms underlying these effects are still under study.

Trinchese et al. [48] tested DM in healthy rat groups taking different isoenergetic diets and observed that the diet supplemented with DM improved glucose disposal and insulin resistance, leading to reduction of glucose levels and better tolerance to glucose loads, compared with the groups not receiving milk-based supplements (control) and taking CM.

Positive effects of DM on glucose metabolism were also observed in rats with streptozocin-induced type 2 diabetes. In this animal model, DM powder supplements reduced blood glucose levels and insulin resistance after four weeks. Remarkably, the anti-diabetic effect of DM was similar to metformin treatment in most biochemical parameters [17].

Along with insulin resistance, pancreatic β-cell dysfunction is typically involved in diabetes development and progression [55]. In this regard, it is noteworthy that DM was able to improve the viability of damaged clonal β-cells (mouse insulinoma β-pancreatic (MIN6) cells) [17].

The DM beneficial effects on glucose metabolism are, at least in part, attributable to:

(1) reduction of inflammatory status and leptin/adiponectin ratio. The animals treated with DM showed a reduction in serum inflammatory mediators and in the leptin/adiponectin ratio [48]. These two hormones, derived from adipocytes, are involved in lipid metabolism, energy homeostasis and inflammation [56,57]. A high leptin to adiponectin ratio is related to insulin resistance [58] and a decrease in adiponectin was found linked to the onset of type 2 diabetes in animal models [59].
(2) enhancement of antioxidant defense mechanisms [17], which protects against the development of insulin resistance.
(3) modulation of mitochondrial dynamics that impacts on mitochondrial metabolism. Alteration of mitochondrial dynamics, function and efficiency has impact on several pathological conditions including metabolic diseases such as obesity and type 2 diabetes [60]. DM-treated rats showed more abundant, larger and electron-dense mitochondria in the skeletal muscle at electron microscopy analysis [49]. These characteristics have been associated to more active mitochondria with higher respiratory capacity and improved glucose metabolism [61].
(4) down-regulation of two gluconeogenesis key enzymes: phosphoenolpyruvate carboxykinase 1 (Pck1) and glucose-6-phosphatase (G6PC) [17].

## 6. Effects on Lipid Metabolism

Some nutritional peculiarities of DM support its use in low-calorie diets and in the management of dyslipidemia. In fact, as previously discussed, DM has a lower calorie and fat concentration (fat ranges from 0.20% to 1.7% in DM) compared with other milks used in human nutrition. In addition, the amount of saturated fatty acids (SFA) in DM is significantly lower than CM (3.02 g/L vs. 26.27 g/L, respectively), while the UFA: SFA ratio is higher (0.75 vs. 0.41, respectively). Furthermore, DM is the richest source of alpha-linolenic acid (C18:3 n-3;ALA) among farm animal milks (about 7.25 g/100 g of fat). C18:3 n-3 ALA is a precursor for long n-3 fatty acids and has beneficial health effects [41]. 

In their controlled studies on murine models, Li et al. and Trinchese et al. [17,48] also found that DM has beneficial effects on lipid metabolism. In DM-fed animals, significantly lowered blood triglycerides and reduced fat accumulation have been observed, which were attributed to beneficial effects on the skeletal muscle. In fact, skeletal muscle mitochondria of DM-fed animals showed increased respiratory capacity and fatty acid oxidation [48].

This effect is due to:

1. An increase in oleylethanolamide (OEA) in the skeletal muscle and in the liver [48]. OEA increase is probably related to the high concentration of palmitic acid in the sn-2 position of the triacylglycerol backbone of DM [5,62]. This type of esterification is similar to that occurring in HM and allows a more effective C16:0 absorption since 2-monoacylglycerols of SFAs are more easily absorbed than free fatty acids (FFA). OEA has been identified as an important regulator of lipid metabolism and can enhance fatty acid oxidation in rats [63].
2. Enhancement of carnitine palmitoyl-transferase (CPT) activity: Increased respiratory capacity in the skeletal muscle is likely related to an enhancement of CPT activity, which would further increase the entry of long-chain FFAs into the mitochondria, stimulating fatty acid oxidation [48]. CPT is a mitochondrial enzyme responsible for the formation of acyl carnitines by catalyzing the transfer of the acyl group of a long-chain fatty acyl-CoA from coenzyme A to l-carnitine. This reaction allows the increase in lipid oxidation for the movement of the acyl carnitine from the cytosol into the intermembrane space of mitochondria.
3. Modulation of mitochondrial function, efficiency, and dynamics: Mitochondrial uncoupling is a dissociation between membrane potential generation and its use for ATP synthesis [64]. Mitochondrial uncoupling dissipates the proton gradient across the inner membrane and creates a futile cycle of glucose and fatty acid oxidation without generating ATP [65], thereby increasing lipid oxidation and reducing intracellular lipid content [66]. Mitochondrial uncoupling induces a less efficient utilization of lipid substrates. This decline in mitochondrial energy efficiency may also contribute to fat burning. Promoting this inefficient metabolism that generates heat instead of ATP, mitochondrial uncoupling can serve as a potential treatment for obesity [64].

## 7. Antiproliferative and Antitumor Effect

The literature contains conflicting reports regarding the relation between the consumption of milk and dairy products and cancer. As regards DM, only a few studies have investigated a possible antiproliferative and antitumor capacity with heterogeneous results.

Indeed, Mao et al. [42] have observed an antiproliferative and antitumor effect of DM WP on A549 human lung cancer cells in a dose-dependent and time-dependent manner. From observations on murine splenocytes, the same authors have concluded that DM WP kill tumor cells through activation of lymphocytes and macrophages.

A recent study in vivo reports that DM reduces primary tumor size and inhibits breast tumor progression in 4T1 mice by inducing apoptosis [67].

According to Esener et al. [68], the release of NO could mediate the DM tumoricidal activity. NO release by PBMCs after DM exposure was also observed by Tafaro et al. [45].

Although the exact role of NO in cancer biology is not fully understood, it seems that high NO concentrations exert a controlling influence on immune-mediated antitumor activities, whereas low concentrations facilitate cell survival and proliferation [69]. On the other hand, NO is a potential oncogenic molecule that promotes neovascularization and reduces blood flow in tumor tissues. Moreover, high concentrations of NO can directly cause DNA damage [68,70].

A randomized study in rats with Ehrlich ascites carcinoma (EAC) found that administration of DM kefir for 10 days reduced the carcinoma volume and increased the number of apoptotic cells compared with the control group [68]. The effects of DM kefir on tumor volume and apoptosis were ascribed to the down-regulation of the NO synthase enzyme (isoforms iNOS and eNOS). In particular, iNOS levels were markedly higher in the control and DM groups compared to the DM kefir group [68]. However, in contrast with the findings of Mao et al. [42] and Li et al. [67], Esener et al. [68] reported that DM was not effective on the carcinoma and the groups of rats treated with unfermented DM showed less numerous apoptotic cells. 

## 8. Protection of the Intestinal Barrier and Modulatory Effect of the Intestinal Flora

Milk oligosaccharides are a complex class of bioactive carbohydrates without direct nutritional value [71].

The nutritional importance of oligosaccharides in milk is due to their prebiotic role (reported for breast milk). In HM, oligosaccharides serve as a substrate for beneficial gut microbiota by acting as prebiotics and inhibit the intestinal adhesion of pathogenic microorganisms, thus limiting the onset of enteric infections. Despite the possible protective role of oligosaccharides on the intestine and the interest in nutritional applications in specific categories of consumers, DM oligosaccharides have been poorly studied [44].

Currently, the results of studies on animal models suggest that DM, and in particular DM LZ, could play a role in the treatment of IBD [46,72,73].

LZ is present in higher quantity in DM compared to ruminant milks and seems quite resistant to human gastrointestinal enzymes (75% resistance) in vitro [74]. Although human studies in infants are still controversial [43], investigations in mouse models show that LZ can reach the intestinal tract intact [72].

Jiang et al. [46] carried out a randomized study on rats gavage fed with fractions of DM containing WP with different percentages of LZ. After 14 days of treatment, colitis was induced in mice (via dextran sulfate sodium). The authors found a protective action of DM WP on the disease: a reduction of symptoms and the improvement of lesions in treated mice compared with controls. The active component responsible for these actions was found to be LZ, while α-LA and β-LG had no significant effects on colitis symptoms. The DM action was linked to a reduction in the mediators of inflammation, but also to the protection of the intestinal mechanical barrier function [46].

Anti‑inflammatory properties of DM on the intestine have been reported in a randomized study on a model of ileitis in mice (ileitis induced by indomethacin) [72]. In this study, oral DM treatment attenuated the severity of symptoms and macroscopic and microscopic damages (drop of body weight, reduction in the length of the small intestine, increase in fecal lipocalin-2). The authors [72] ascribed these actions to the normalization of the intestinal immunity function, in particular to the expression of antimicrobial peptides by the Paneth cells which contribute directly to reduce dysbiosis.

Both raw and thermized DM counteracted chronic stress-induced intestinal damage, gut hyper-permeability and inflammation in mouse models of chronic stress [73]. Yvon et al. [73] highlight the importance of DM LZ activity in the reduction of intestinal damage. 

DM was also found to modulate the intestinal microbiota and increase the microbial diversity in healthy rats [49] and in mouse models with IBD [46]. A positive modulation of the microbiota was also observed in the study of Yvon et al. [72], though this effect was linked to indirect actions on the intestinal immunity of the host rather than direct actions on the microflora.

According to Penders et al. [75], the beneficial effects of DM on the microbiota could represent a key source of immune development and regulation in early life and could have a preventive role against the development of atopic dermatitis.

**Table 3:** In vitro studies on the beneficial effects of donkey milk.

Experimental Model	Effects	Reference
Mouse insulinoma beta-pancreatic (MIN6) cells	Anti-diabetes action: DM in the medium (500 μg/mL) improved the viability of damaged pancreatic beta-cells	[17]
DM ^1^ and fermented DM samples	Antioxidant activity of fermented DM samples Antihypertensive effect (ACE-inhibitory activity) in fermented DM	[18]
Murine splenocytes	Immunological modulation: increase in IL-1, IL-6, TNF-α, IL-2 and IFN-γ	[42]
A549 human lung cancer cells	Anti-proliferative activity induced by DM whey protein (MW ^2^ > 10 kDa),	
Human peripheral blood mononuclear cells	Immunological activities: increase in IL-1 and IL-10	[45]

^1^ DM: donkey milk; ^2^ MW: molecular weight.

**Table 4:** In vivo randomized controlled studies on the beneficial effects of donkey milk.

Experimental Model	Treatment	Effects	Reference
Balb/c mice with induced colitis	3 DM ^1^ whey fraction (5%, 20% and 50% of lysozyme) for 14 days	Immunological activities: inhibition of IL-13 and TNF-α Improvement in the intestinal barrier and modulatory effects on the gut microbiota.	[46]
14 elderly subjects ( from 72 to 97 years old)	200 mL/day of DM for one month	Immunological activities: increase in IL-1, IL-6 and TNF-α	[47]
Wistar rats	3 g/kg day of DM powder for 4 weeks.	Antioxidant effects: tendency to increase SOD ^2^ activity in the plasma of diabetic rats	[17]
		Improvement of metabolism: Reduction in the blood glucose on type 2 diabetic rats and in insulin resistance	
Wistar rats	48 mL/day of DM, for 4 weeks	Antioxidant effects: improvements in oxidative stress markers in the liver; increased activities of liver detoxifying enzymes, increase of antioxidants	[48,49]
		Improvement of metabolism: improved glucose disposal; decrease of blood triglycerides and of fat accumulation in muscles; modulation of the intestinal microbiota	
Swiss albino mice. with Ehrlich ascites carcinoma tumour	0.5 mL/day of DM or kefir of DM for 10 days	Anti-proliferative activity: reduction in tumor volume and increased number of apoptotic cells in the groups treated with fermented DM, not in the groups treated with unfermented DM	[68]
C57BL/6 mice ileitis induced	Orally treated with DM with the same total daily activity of lysozyme, i.e., 11800 UI in a total adjusted volume of 0.4 (± 0.05) mL for 7 days	Reduction of dysbiosis by mean of stimulation of the intestinal innate immunity	[72]

^1^ DM: donkey milk; ^2^ SOD: superoxide dis-mutase.

## 9. Antibacterial Properties

DM showed antibacterial properties that may be linked to a synergistic activity of LZ, lactoferrin and some FFA such us lauric, oleic and linoleic acids [76,77]. 

Lactoferrin exhibits antibacterial, antiviral, antifungal and antiprotozoal activities [39]. Differently from HM, lactoferrin is a minor component in DM (0.097–0.133 g/L), has poor thermal resistance [78] and is easily digested by gastric and duodenal juice. Thus, it has been suggested that lactoferrin can play its biologic role in vivo mainly through its bioactive peptides derived from digestion [79].

In addition to lactoferrin, α-LA, highly concentrated in DM, may contribute to inhibit the growth of potential pathogens, as reported both in vitro and in vivo for the human protein [39].

The antibacterial effect of DM could also be mediated by the microflora of the milk itself. DM contains *Lactobacillus plantarum* that was described to produce bactericidal bacteriocins [80]. In addition, antimicrobial activities were found to be higher in fermented than raw DM and were further increased after in vitro simulated gastrointestinal digestion [18], suggesting that the formation of bioactive peptides may play an additional role in this effect.

Several authors confirm the efficacy of DM in inhibiting the growth of specific foodborne pathogens in vitro (Table 5), specifically Gram + bacteria such as:

(a) *Listeria monocytogenes* (2230/92, ATCC 19111; ATCC: 13932), which was inhibited at concentration of 1% by DM, in vitro digested DM [19] and also in situ, in artificially contaminated milk [76,81].
(b) *Staphylococcus aureus* (ATCC 8095; ATCC: 6538). DM (50 folds concentrated) was active at minimal lethal concentration of 64 mg/mL [82], also in situ [76,81,83]. However, some authors [82,84] found that this antimicrobial activity is reduced by digestion.
(c) *Enterococcus faecalis* (DSM 2352), which was inhibited by hydrolyzed DM [84].

**Table 5:** Studies on effects against microorganisms of donkey milk.

Microorganism	Experimental Model	Reference
*Listeria monocytogenes* (2230/92; ATCC 19111; ATCC: 13932)	digested in vitro DM ^1^ and DM at concentration 1% on microtiter plates;	[19]
	in situ	[76,81]
*Staphylococcus aureus* (ATCC 8095) (minimal lethal concentration of 64 mg of DM concentrated to 50 folds/mL)	agar well diffusion	[82]
*Staphylococcus aureus* (ATCC 8095 ATCC 25923 ATCC: 6538) and (DSM 25923) (by hydrolyzed DM)	in situ	[76,81,83,84]
*Enterococcus faecalis* (DSM 2352)	hydrolyzed DM milk tested by inhibition halos test on agar plates	[84]
*Salmonella enterica serovar choleraesuis* (CGMCC 1.1859)	Agar diffusion test	[85]
*Salmonella serovar enteritidis* (ATCC 13076). and *serovar Typhimurium* (ATCC 14028)	In situ	[81,83];
*Shigella dysenteriae*(CGMCC 1.1869)	agar diffusion test and in situ	[85]
*Microsporum canis, and Microsporum gypseum* (failed to grow at a concentration of 60% and 70% of donkey milk respectively)	microdilution test	[77]
*Trichophyton mentagrophytes and T. rubrum* (minimal lethal concentration 32 mg of 50 folds concentrated DM/mL)	agar well diffusion test	[82]
Echovirus type 5	1 mg of DM WP^2^ fractions/mL in the medium of growth of infected culture of human intestinal epithelial cell line Caco-2	[84]

^1^ DM: donkey milk; WP: whey protein.

Relating to *Bacillus cereus*, DM showed variable efficacy depending on the strain [82,84]. For example, DM was less active against B. cereus DSM 4384 [84], while *B. cereus* RT INF01 appeared very resistant [19].

An antibacterial activity was also reported against the Gram—*Salmonella enterica serovar choleraesuis* (CGMCC 1.1859), *serovar enteriti*dis (ATCC 13076) and *serovar Typhimurium* (ATCC 14028). DM was active against these bacteria on the agar diffusion test [85] and in artificially contaminated milk [81,83]. *Shigella dysenteriae* (CGMCC 1.1869) was also sensitive to DM on the agar diffusion test and in situ. In fact, the counts of viable *S. dysenteriae* decreased to below detectable levels in artificially contaminated milk [85].

Regarding *Escherichia coli*, the results in the literature are conflicting and probably related to a strain-dependent activity. In fact, some authors [19] showed that DM and in vitro digested DM caused growth reduction on *E. coli* (EPEC) 10208355 during its stationary phase at concentrations of 0.6 and 1.0%. Moderate antibacterial activities against *E. coli* strain ATCC 25922 were also observed by Koutb et al. [82] with milk concentrated 50 times (minimal lethal concentrations of 128 mg/mL). In studies in situ on artificially contaminated milk, an inhibitory activity of DM on the development of *E. coli* has been reported [83]. Differently, the growth of the strain C84010 was not inhibited by DM on agar diffusion assay [85], and the toxicogenic *E. coli* DSM 8579 was very resistant to in vitro digested DM [84].

## 10. Antifungal and Antiviral Properties

DM proved to be inhibitory against fungi (Table 5), particularly some dermatophytes [77,82], with the potential to prevent and control the infection of these zoonotic fungi in humans.

*Microsporum canis*, and *Trichophyton mentagrophytes* failed to grow in 60% DM and *Microsporum gypseum* appeared to be sensitive to 70% DM [77]. 

Also, Koutb et al. [82] reported a minimal lethal concentration of 32 mg/mL against *T. mentagrophytes* and *T. rubrum*, using a 50-fold concentrated milk, while no growth inhibition was observed when testing *Candida albicans*. The activity against dermatomycotic fungi was not affected after digestion of DM with pepsin by Koutb et al. [82].

The only paper that investigated the antiviral activity of the DM reports its effectiveness against Echovirus (Enteric Cytopathic Human Orphan virus) Type 5 [86]. Echovirus is a small, non-enveloped, single stranded RNA virus, belonging to the genus Enterovirus of the Picornaviridae family, acquired by fecal–oral contamination, and infecting the gastrointestinal tract as the primary organ [87]. Infections with echoviruses have been associated with a wide variety of neurological and exanthematic diseases [88].

Among the different protein fractions of DM tested on human intestinal epithelial cell lines (Caco-2) infected with Echovirus Type 5, WP showed the greatest inhibition on virus replication [86]. In particular, DM antiviral activity on echovirus type 5 seems due to a synergic action of high molecular mass WP, such as lactoferrin, lactoperoxidase, serum albumin, and immunoglobulins [86].

## 11. Conclusions

Several clinical studies report that DM shows high tolerability in children with food allergies, while DM immunomodulatory properties have been described in animal models and in a single study in humans. Research on murine models shows that DM modulates the intestinal microbiota, enhances antioxidant defense mechanisms and detoxifying enzymes, and is effective in controlling blood sugar and dyslipidemias. Although the first in vitro study on the antiproliferative and antitumor effect of DM yielded promising results, the few available trials in animal models show conflicting findings. Finally, in vitro studies describe inhibitory actions of DM on bacteria, viruses and fungi. So far, the observed beneficial properties of DM have been tested almost exclusively in vitro and in animal models and have been mostly related to some WP. From this literature review, there emerges a need for new randomized clinical trials on DM consumption in humans to provide stronger evidence of its potential beneficial health effects, which could lead to new applications of DM as an adjuvant in human medicine.

## Data Availability

Not applicable.

## References

[1] Adams F. (1922). The Genuine Works of Hippocrates. South. Med. J..

[2] (1855). Pliny the Elder. The Natural History. Book XXVIII “Remedies Derived from Living Creatures”.

[3] Lu D.L., Zhang D.F., Liu P.L., Dong M.L. (2006). The nutrition value and exploitation of donkey milk. J. Dairy Sci. Technol..

[4] Lauzier A.-C. (2011). Pratiques D’allaitement à Port-Royal et Aux Enfants-Assistés à la Fin du XIXe Siècle. Gynécologie et Obstétrique. https://dumas.ccsd.cnrs.fr/dumas-00625364.

[5] Altomonte I., Salari F., Licitra R., Martini M. (2019). Donkey and human milk: Insights into their compositional similarities. Int. Dairy J..

[6] Massouras T., Triantaphyllopoulos K.A., Theodossiou I. (2017). Chemical composition, protein fraction and fatty acid profile of donkey milk during lactation. Int. Dairy J..

[7] Salgado M.J.G., Silva A.R., de Souza C.O., Lemos P.V.F., Oliveira R.L., Oliveira C.A.D.A., Ribeiro C.V.D.M. (2021). Days in milk alters the milk fatty acid profile of grazing donkeys: A preliminary study. J. Anim. Physiol. Anim. Nutr..

[8] Martini M., Salari F., Licitra R., La Motta C., Altomonte I. (2019). Lysozyme activity in donkey milk. Int. Dairy J..

[9] Licitra R., Chessa S., Salari F., Gattolin S., Bulgari O., Altomonte I., Martini M. (2019). Milk protein polymorphism in Amiata donkey. Livest. Sci..

[10] Martini M., Altomonte I., Licitra R., Salari F. (2018). Short communication: Technological and seasonal variations of vitamin D and other nutritional components in donkey milk. J. Dairy Sci..

[11] Vincenzetti S., Pucciarelli S., Santini G., Klimanova Y., Polzonetti V., Polidori P. (2020). B-Vitamins Determination in Donkey Milk. Beverages.

[12] Calvano C.D., Glaciale M., Palmisano F., Cataldi T.R. (2018). Glycosphingolipidomics of donkey milk by hydrophilic interaction liquid chromatography coupled to ESI and multistage MS. Electrophoresis.

[13] Martini M., Altomonte I., Licitra R., Bartaloni F., Salari F. (2021). A preliminary investigation into the unsaponifiable fraction of donkey milk: Sterols of animal origin, phytosterols, and tocopherols. J. Dairy Sci..

[14] Lazarevic J., Tasic T., Popovic S., Banjac V., Djuragic O., Kokic B., Cabarkapa I. (2017). Changes in milk composition of domestic Balkan donkeys’ breed during lactation periods. Acta Period. Technol..

[15] Li W., Li M., Cao X., Han H., Kong F., Yue X. (2020). Comparative analysis of whey proteins in donkey colostrum and mature milk using quantitative proteomics. Food Res. Int..

[16] Cunsolo V., Saletti R., Muccilli V., Gallina S., Di Francesco A., Foti S. (2017). Proteins and bioactive peptides from donkey milk: The molecular basis for its reduced allergenic properties. Food Res. Int..

[17] Li Y., Fan Y., Shaikh A.S., Wang Z., Wang D., Tan H. (2020). Dezhou donkey (Equus asinus) milk a potential treatment strategy for type 2 diabetes. J. Ethnopharmacol..

[18] Aspri M., Leni G., Galaverna G., Papademas P. (2018). Bioactive properties of fermented donkey milk, before and after in vitro simulated gastrointestinal digestion. Food Chem..

[19] Tidona F., Sekse C., Criscione A., Jacobsen M., Bordonaro S., Marletta D., Vegarud G.E. (2011). Antimicrobial effect of donkeys’ milk digested in vitro with human gastrointestinal enzymes. Int. Dairy J..

[20] Tidona F., Criscione A., Devold T.G., Bordonaro S., Marletta D., Vegarud G.E. (2014). Protein composition and micelle size of donkey milk with different protein patterns: Effects on digestibility. Int. Dairy J..

[21] Mignone L.E. (2015). Whey protein: The “whey” forward for treatment of type 2 diabetes?. World J. Diabetes.

[22] Barni S., Sarti L., Mori F., Muscas G., Belli F., Pucci N., Novembre E. (2018). Tolerability and palatability of donkey’s milk in children with cow’s milk allergy. Pediatr. Allergy Immunol..

[23] Monti G., Bertino E., Muratore M.C., Coscia A., Cresi F., Silvestro L., Fabris C., Fortunato D., Giuffrida M.G., Conti A. (2007). Efficacy of donkey’s milk in treating highly problematic cow’s milk allergic children: An in vivo and in vitro study. Pediatr. Allergy Immunol..

[24] Monti G., Viola S., Baro C., Cresi F., Tovo P.A., Moro G., Ferrero M.P., Conti A., Bertino E. (2012). Tolerability of donkey’s milk in 92 highly problematic cow’s milk allergic children. J. Biol. Regul. Homeost. Agents.

[25] Sarti L., Martini M., Brajon G., Barni S., Salari F., Altomonte I., Ragona G., Mori F., Pucci N., Muscas G. (2019). Donkey’s Milk in the Management of Children with Cow’s Milk protein allergy: Nutritional and hygienic aspects. Ital. J. Pediatr..

[26] Vita D., Passalacqua G., Di Pasquale G., Caminiti L., Crisafulli G., Rulli I., Pajno G.B. (2007). Ass’s milk in children with atopic dermatitis and cow’s milk allergy: Crossover comparison with goat’s milk. Pediatr. Allergy Immunol..

[27] Mori F., Sarti L., Barni S., Pucci N., Belli F., Stagi S., Novembre E. (2017). Donkey´s Milk Is Well Accepted and Tolerated by Infants With Cow´s Milk Food Protein–Induced Enterocolitis Syndrome: A Preliminary Study. J. Investig. Allergol. Clin. Immunol..

[28] Peeters C., Herman A., Baeck M. (2017). Donkey’s milk allergy. Br. J. Dermatol..

[29] Giorgis V., Rölla G., Raie A., Geuna M., Boita M., Lamberti C., Nebbia S., Giribaldi M., Giuffrida M., Brussino L. (2018). A Case of Work-Related Donkey Milk Allergy. J. Investig. Allergol. Clin. Immunol..

[30] Martini M., Swiontek K., Antonicelli L., Garritani M.S., Bilò M.B., Mistrello G., Amato S., Revets D., Ollert M., Morisset M. (2018). Lysozyme, a new allergen in donkey’s milk. Clin. Exp. Allergy.

[31] Tesse R., Paglialunga C., Braccio S., Armenio L. (2009). Adequacy and tolerance to ass’s milk in an Italian cohort of children with cow’s milk allergy. Ital. J. Pediatr..

[32] Aranceta J., Pérez-Rodrigo C. (2012). Recommended dietary reference intakes, nutritional goals and dietary guidelines for fat and fatty acids: A systematic review. Br. J. Nutr..

[33] Fiocchi A., Brozek J., Schünemann H., Bahna S.L., von Berg A., Beyer K., Bozzola M., Bradsher J., Compalati E., Ebisawa M. (2010). World Allergy Organization (WAO) Diagnosis and Rationale for Action against Cow’s Milk Allergy (DRACMA) Guidelines. World Allergy Organ. J..

[34] Muraro M.A., Giampietro P.G., Galli E. (2002). Soy formulas and nonbovine milk. Ann. Allergy, Asthma Immunol..

[35] Bertino E., Cavallarin L., Cresi F., Tonetto P., Peila C., Ansaldi G., Raia M., Varalda A., Giribaldi M., Conti A. (2019). A Novel Donkey Milk–derived Human Milk Fortifier in Feeding Preterm Infants: A Randomized Controlled Trial. J. Pediatr. Gastroenterol. Nutr..

[36] Cresi F., Maggiora E., Pirra A., Tonetto P., Rubino C., Cavallarin L., Giribaldi M., Moro G.E., Peila C., Coscia A. (2020). Effects on Gastroesophageal Reflux of Donkey Milk-Derived Human Milk Fortifier Versus Standard Fortifier in Preterm Newborns: Additional Data from the FortiLat Study. Nutrients.

[37] Peila C., Spada E., DeAntoni S., Iuliano E., Moro G.E., Giribaldi M., Cavallarin L., Cresi F., Coscia A. (2020). The “Fortilat” Randomized Clinical Trial Follow-Up: Neurodevelopmental Outcome at 18 Months of Age. Nutrients.

[38] Corvaglia L., Zama D., Gualdi S., Ferlini M., Aceti A., Faldella G. (2008). Gastro-oesophageal reflux increases the number of apnoeas in very preterm infants. Arch. Dis. Child. Fetal Neonatal Ed..

[39] Lönnerdal B., Erdmann P., Thakkar S.K., Sauser J., Destaillats F. (2017). Longitudinal evolution of true protein, amino acids and bioactive proteins in breast milk: A developmental perspective. J. Nutr. Biochem..

[40] Addo C.N.A., Ferragut V. (2015). Evaluating the Ultra-High Pressure Homogenization (UHPH) and Pasteurization effects on the quality and shelf life of donkey milk. Int. J. Food Stud..

[41] Martini M., Altomonte I., Licitra R., Salari F. (2018). Nutritional and Nutraceutical Quality of Donkey Milk. J. Equine Vet. Sci..

[42] Mao X., Gu J., Sun Y., Xu S., Zhang X., Yang H., Ren F. (2009). Anti-proliferative and anti-tumour effect of active components in donkey milk on A549 human lung cancer cells. Int. Dairy J..

[43] Lönnerdal B., Forsum E., Hambraeus L. (1977). A Longitudinal Study of the Protein Content of Human Milk from Well-Nourished Swedish Mothers. Ann. Nutr. Metab..

[44] Licitra R., Li J., Liang X., Altomonte I., Salari F., Yan J., Martini M. (2019). Profile and content of sialylated oligosaccharides in donkey milk at early lactation. LWT.

[45] Tafaro A., Magrone T., Jirillo F., Martemucci G., D’Alessandro A., Amati L. (2007). Immunological Properties of Donkeys Milk: Its Potential Use in the Prevention of Atherosclerosis. Curr. Pharm. Des..

[46] Jiang L., Lv J., Liu J., Hao X., Ren F., Guo H. (2018). Donkey milk lysozyme ameliorates dextran sulfate sodium-induced colitis by improving intestinal barrier function and gut microbiota composition. J. Funct. Foods.

[47] Amati L., Marzulli G., Martulli M., Tafaro A., Jirillo F., Pugliese V., Martemucci G., D’Alessandro A. (2010). Donkey and Goat Milk Intake and Modulation of the Human Aged Immune Response. Curr. Pharm. Des..

[48] Trinchese G., Cavaliere G., De Filippo C., Aceto S., Prisco M., Chun J.T., Penna E., Negri R., Muredda L., Demurtas A. (2018). Human Milk and Donkey Milk, Compared to Cow Milk, Reduce Inflammatory Mediators and Modulate Glucose and Lipid Metabolism, Acting on Mitochondrial Function and Oleylethanolamide Levels in Rat Skeletal Muscle. Front. Physiol..

[49] Trinchese G., Cavaliere G., Canani R.B., Matamoros S., Bergamo P., De Filippo C., Aceto S., Gaita M., Cerino P., Negri R. (2015). Human, donkey and cow milk differently affects energy efficiency and inflammatory state by modulating mitochondrial function and gut microbiota. J. Nutr. Biochem..

[50] Piovesana S., Capriotti A.L., Cavaliere C., La Barbera G., Samperi R., Chiozzi R.Z., Laganà A. (2015). Peptidome characterization and bioactivity analysis of donkey milk. J. Proteom..

[51] Chiozzi R.Z., Capriotti A.L., Cavaliere C., La Barbera G., Piovesana S., Samperi R., Laganà A. (2016). Purification and identification of endogenous antioxidant and ACE-inhibitory peptides from donkey milk by multidimensional liquid chromatography and nanoHPLC-high resolution mass spectrometry. Anal. Bioanal. Chem..

[52] Ugidos-Rodríguez S., Matallana-González M.C., Sánchez-Mata M.C. (2018). Lactose malabsorption and intolerance: A review. Food Funct..

[53] Kaskous S. (2021). Cow’s milk consumption and risk of disease. Emir. J. Food Agric..

[54] Nilsson M., Stenberg M., Frid A.H., Holst J.J., Björck I.M.E. (2004). Glycemia and insulinemia in healthy subjects after lactose-equivalent meals of milk and other food proteins: The role of plasma amino acids and incretins. Am. J. Clin. Nutr..

[55] Weng J., Ji L., Jia W., Lu J., Zhou Z., Zou D., Zhu D., Chen L., Chen L., Guo L. (2016). Standards of care for type 2 diabetes in China. Diabetes/Metab. Res. Rev..

[56] Minokoshi Y., Kim Y.-B., Peroni O.D., Fryer L.G.D., Müller C., Carling D., Kahn B.B. (2002). Leptin stimulates fatty-acid oxidation by activating AMP-activated protein kinase. Nat. Cell Biol..

[57] Yamauchi T., Kamon J., Minokoshi Y., Ito Y., Waki H., Uchida S., Yamashita S., Noda M., Kita S., Ueki K. (2002). Adiponectin stimulates glucose utilization and fatty-acid oxidation by activating AMP-activated protein kinase. Nat. Med..

[58] Oda N., Imamura S., Fujita T., Uchida Y., Inagaki K., Kakizawa H., Hayakawa N., Suzuki A., Takeda J., Horikawa Y. (2008). The ratio of leptin to adiponectin can be used as an index of insulin resistance. Metabolism.

[59] Chakraborti C.K. (2015). Role of adiponectin and some other factors linking type 2 diabetes mellitus and obesity. World J. Diabetes.

[60] Cavaliere G., Trinchese G., Bergamo P., De Filippo C., Mattace Raso G., Gifuni G., Putti R., Moni B.H., Canani R.B., Meli R. (2016). Polyunsaturated Fatty Acids Attenuate Diet Induced Obesity and Insulin Resistance, Modulating Mitochondrial Respiratory Uncoupling in Rat Skeletal Muscle. PLoS ONE.

[61] Mannella C.A. (2006). The relevance of mitochondrial membrane topology to mitochondrial function. Biochim. Biophys. Acta.

[62] Innis S.M. (2015). Palmitic Acid in Early Human Development. Crit. Rev. Food Sci. Nutr..

[63] Li L., Li L., Chen L., Lin X., Xu Y., Ren J., Fu J., Qiu Y. (2015). Effect of oleoylethanolamide on diet-induced nonalcoholic fatty liver in rats. J. Pharmacol. Sci..

[64] Demine S., Renard P., Arnould T. (2019). Mitochondrial Uncoupling: A Key Controller of Biological Processes in Physiology and Diseases. Cells.

[65] Tseng Y.-H., Cypess A.M., Kahn C.R. (2010). Cellular bioenergetics as a target for obesity therapy. Nat. Rev. Drug Discov..

[66] Harper M.-E., Green K., Brand M.D. (2008). The Efficiency of Cellular Energy Transduction and Its Implications for Obesity. Annu. Rev. Nutr..

[67] Li Q., Li M., Zhang J., Shi X., Yang M., Zheng Y., Cao X., Yue X., Ma S. (2020). Donkey milk inhibits triple-negative breast tumor progression and is associated with increased cleaved-caspase-3 expression. Food Funct..

[68] Esener O., Balkan B., Armutak E., Uvez A., Yildiz G., Hafizoglu M., Yilmazer N., Gurel-Gurevin E. (2018). Donkey milk kefir induces apoptosis and suppresses proliferation of Ehrlich ascites carcinoma by decreasing iNOS in mice. Biotech. Histochem..

[69] Vannini F., Kashfi K., Nath N. (2015). The dual role of iNOS in cancer. Redox Biol..

[70] Taskin E.I., Kapucu A., Akgün-Dar K., Yagci A., Caner M., Firat U.B., Firat I., Dogruman H. (2008). Effects of an oral treatment with concentrated grape juice (ENT) on cell NOS (eNOS and iNOS) expression and proliferation in experimentally induced carcinoma in mice. Rev. Méd. Vét..

[71] Doherty A.M., Lodge C.J., Dharmage S.C., Dai X., Bode L., Lowe A.J. (2018). Human Milk Oligosaccharides and Associations With Immune-Mediated Disease and Infection in Childhood: A Systematic Review. Front. Pediatr..

[72] Yvon S., Olier M., Leveque M., Jard G., Tormo H., Haimoud-Lekhal D.A., Peter M., Eutamène H. (2018). Donkey milk consumption exerts anti-inflammatory properties by normalizing antimicrobial peptides levels in Paneth’s cells in a model of ileitis in mice. Eur. J. Nutr..

[73] Yvon S., Schwebel L., Belahcen L., Tormo H., Peter M., Haimoud-Lekhal D.A., Eutamene H., Jard G. (2019). Effects of thermized donkey milk with lysozyme activity on altered gut barrier in mice exposed to water-avoidance stress. J. Dairy Sci..

[74] Marletta D., Tidona F., Bordonaro S., Gigli I. (2016). Donkey Milk Proteins: Digestibility and Nutritional Significance. Milk Proteins—From Structure to Biological Properties and Health Aspects.

[75] Penders J., Gerhold K., Stobberingh E.E., Thijs C., Zimmermann K., Lau S., Hamelmann E. (2013). Establishment of the intestinal microbiota and its role for atopic dermatitis in early childhood. J. Allergy Clin. Immunol..

[76] Saric L., Saric B., Kravic S., Plavsic D., Milovanovic I., Gubić J.M., Nedeljković N.M. (2014). Antibacterial activity of domestic Balkan donkey milk toward Listeria monocytogenes and Staphylococcus aureus. Food Feed. Res..

[77] Altomonte I., Nardoni S., Mancianti F., Perrucci S., Licitra R., Salari F., Martini M. (2019). Preliminary results on antifungal activity of donkey milk. Large Anim. Rev..

[78] Ozturkoglu-Budak S. (2018). Effect of different treatments on the stability of lysozyme, lactoferrin and β-lactoglobulin in donkey’s milk. Int. J. Dairy Technol..

[79] Brumini D., Criscione A., Bordonaro S., Vegarud G.E., Marletta D. (2016). Whey proteins and their antimicrobial properties in donkey milk: A brief review. Dairy Sci. Technol..

[80] Murua A., Todorov S., Vieira A., Martinez R., Cencič A., Franco B. (2013). Isolation and identification of bacteriocinogenic strain of Lactobacillus plantarum with potential beneficial properties from donkey milk. J. Appl. Microbiol..

[81] Ebrahimi A., Moosavy M., Khatibi S.A., Barabadi Z., Hajibemani A. (2021). A comparative study of the antibacterial properties of milk from different domestic animals. Int. J. Dairy Technol..

[82] Koutb M., Khider M., Ali E.H., Hussein N.A. (2016). Antimicrobial Activity of Donkey Milk against Dermatomycotic Fungi and Foodborne Bacteria. Int. J. Biomed. Mater. Res..

[83] Šarić L.Ć., Šarić B.M., Mandić A.I., Torbica A.M., Tomić J.M., Cvetković D.D., Okanović Đ.G. (2012). Antibacterial properties of Domestic Balkan donkeys’ milk. Int. Dairy J..

[84] Nazzaro F. (2010). Isolation of Components with Antimicrobial Property from the Donkey Milk: A Preliminary Study. Open Food Sci. J..

[85] Zhang X.-Y., Zhao L., Jiang L., Dong M.-L., Ren F.-Z. (2008). The antimicrobial activity of donkey milk and its microflora changes during storage. Food Control..

[86] Brumini D., Furlund C.B., Comi I., Devold T.G., Marletta D., Vegarud G.E., Jonassen C.M. (2013). Antiviral activity of donkey milk protein fractions on echovirus type 5. Int. Dairy J..

[87] Tinari A., Pietrantoni A., Ammendolia M.G., Valenti P., Superti F. (2005). Inhibitory activity of bovine lactoferrin against echovirus induced programmed cell death in vitro. Int. J. Antimicrob. Agents.

[88] Park K., Song J., Baek K., Lee C., Kim D., Cho S., Park J., Choi Y., Kang B., Choi H. (2011). Genetic diversity of a Korean echovirus 5 isolate and response of the strain to five antiviral drugs. Virol. J..

